# Expression of cytokines in dairy cattle mammary gland parenchyma during chronic staphylococcal infection

**DOI:** 10.1186/s13567-021-01003-y

**Published:** 2021-10-18

**Authors:** Ewelina Kawecka-Grochocka, Magdalena Zalewska, Magdalena Rzewuska, Ewa Kościuczuk, Tomasz Ząbek, Tomasz Sakowski, Sylwester Marczak, Emilia Bagnicka

**Affiliations:** 1 Department of Biotechnology and Nutrigenomics, Institute of Genetics and Animal Biotechnology PAS, Postepu 36A, 05-552 Jastrzębiec, Poland; 2 Department of Preclinical Sciences, Faculty of Veterinary Medicine, Warsaw University of Life Sciences SGGW, Nowoursynowska 166f, 02-787 Warsaw, Poland; 3 Department of Bacterial Physiology, Institute of Microbiology, Faculty of Biology, University of Warsaw, Miecznikowa 1, 02-096 Warsaw, Poland; 4 Department of Animal Molecular Biology, National Research Institute of Animal Production, Krakowska 1, 32-083 Balice, Poland; 5 Present Address: Robert H. Lurie Comprehensive Cancer Center, Northwestern University, Chicago, IL 60611 USA

**Keywords:** *Bos taurus*, udder, chronic mastitis, bacterial infection, CoPS, CoNS, immune response, mammary gland, parenchyma

## Abstract

The study aim was to determine the expression of genes potentially related to chronic mastitis at the mRNA and protein levels, viz*.* chemokine C–C motif receptor 1 (*CCR1*), C–C motif chemokine ligand 2 (*CCL2*), C–C motif chemokine ligand 5 (*CXCL5*), tumor necrosis factor α (*TNFα*), interleukin 1β (*IL-1β*), interleukin 6 (*IL-6*), interleukin 8 (*IL-8*), interleukin 18 (*IL-18*), in bovine mammary gland parenchyma. The study examines the differences in expression of selected genes between cows with chronic mastitis caused by coagulase-positive (CoPS) or coagulase-negative staphylococci (CoNS) and those with healthy udders (H). Samples were collected from the udder quarters from 40 Polish Holstein–Friesian cows; 54 of these samples were chosen for analysis based on microbiological analysis of milk taken two days before slaughter. They were categorized into three groups: CoPS (*N* = 27), CoNS (*N* = 14) and H (*N* = 13). The RNA expression was analyzed by RT-qPCR and protein concentration by ELISA. No differences in the mRNA levels of seven genes (*TNFα*, *IL-18*, *CCR1*, *IL-1β*, *CCL2*, *IL-8*, *IL-6*) and four proteins (TNFα, IL-18, CCR1, IL-1β) were identified between the CoPS and H groups. Higher transcript levels of *CXCL5* (*p* ≤ 0.05) gene were noted in CoPS than in H. Compared to H, higher concentrations of IL-8 and CXCL5 (*p* ≤ 0.05) were observed in CoPS (0.05 < *p* < 0.1) and CCL2 (0.05 < *p* < 0.1) in CoNS, while lower levels of Il-6 were found in CoPS. This may suggest that during chronic mastitis the organism stops producing pro-inflammatory cytokines, probably to protect the host tissues against their damage during prolonged infection.

## Introduction

Mastitis is the most common and, hence, the most cost-generating disease in the dairy cattle industry worldwide. Invasion of the udder by pathogenic microorganisms through the teat canal results in various physical, chemical and microbiological changes in milk. The most commonly isolated bacteria from mastitic bovine milk are staphylococci. Traditionally, two groups of mastitis-causing staphylococci have been recognized based on their ability to produce coagulase, an enzyme responsible for converting fibrinogen into fibrin: these are coagulase-positive staphylococci (CoPS) and coagulase-negative staphylococci (CoNS). Typically, CoPS are considered as major pathogens, while CoNS as environmental, minor pathogens; however, some CoNS have also recently been found to produce toxins and cause severe mastitis [1]. Moreover, bacterial infection can stimulate the immune response to different degrees, and this can determine the type of resulting inflammation, i.e. clinical vs*.* subclinical infection. The first line of the non-specific defense used by the host, apart from the physical and chemical barrier, is the innate immune system; this comprises a complex system of macrophages, neutrophils, or natural killer cells (NK), and a number of non-specific proteins such as the complement system, defensins, lactoferrin, or cathepsin. The non-specific immune reaction as a whole comprises the detection of pathogens and host tissue damage, removal of pathogens and repair of host tissue, followed by activating the mechanisms of the specific immune reaction [2].

The first step of adaptive (specific) immunity is guided by pathogen-recognition receptors (PPR) as mediators of inflammation and immune response. They can be present on the surface of immune cells (membrane-bound PPR), or as secreted and locally-produced molecules (cytoplasmic PPR). They recognize pathogen-associated molecular patterns (PAMP) present on the pathogen cell wall, such as lipopolysaccharide (LPS) or teichoic acid (LTA) [3]. One group of PPR are toll-like receptors (TLR) recognizing pathogens with high specificity; the binding of TLR to the PAMP stimulates the activation of phagocytosis and pathogen killing and the release of anti-microbial peptides and pro-inflammatory cytokines, as a part of the non-specific immune response. Adaptive immunity employs mechanisms that rely on antigen presentation, and its response is triggered when the innate response fails. The division of resistance into specific and non-specific is to some extent conventional, because in fact only their joint action gives a chance to fight the pathogen. The susceptibility or resistance of the host to infection is determined by the intensity of activity of both types of immunity [4].

One group of proteins involved in the innate immunity system are cytokines: small (10–50 kDa) molecules with similar activity to hormones and which are named according to the place of secretion, e.g. lymphokines are produced by lymphocytes and monokines by monocytes, or according to their function, e.g. chemokines exhibit chemotactic activity. Cytokines may affect cells that produce/secrete them (autocrine mode of action), as well as nearby cells (paracrine manner of action), or cells in other organs (endocrine mode of action) [5]. When defending the mammary gland tissue, NK cells recognize the pathogen and trigger an inflammatory response by the expression of certain cytokines, e.g. tumor necrosis factor α (TNFα), interleukin 1β (IL-1β), interleukin 8 (IL-8); these recruit neutrophils and macrophages from the bloodstream to the site of inflammation. Cytokines may also increase the phagocytic activity of macrophages and neutrophils [6]. Microarray studies and Kyoto Encyclopedia of Genes and Genomes (KEGG) analysis by Kościuczuk et al. [7] found the *chemokine signaling pathway* and the *cytokine receptor interaction pathway* to be over represented during infection.

The cytokine network is not yet fully elucidated, and despite the vast number of in vivo studies on bovine udder inflammation, the processes in the chronic anti-inflammation response remain unclear. Therefore, it is crucial to study this mechanism on the molecular level to better understand the components of an effective antibacterial response. The aim of the present study is to determine the expression of eight genes known to participate in the immune response in bovine mammary gland secretory tissue (MGST) at the transcript and protein levels in a model of chronic mastitis, and to compare the findings with the expression observed in healthy tissue. The study focused on chemokine C–C motif receptor 1 (CCR1), C–C motif chemokine ligand 2 (CCL2), C–C motif chemokine ligand 5 (CXCL5), TNFα, IL-1β, interleukin 6 (IL-6), IL-8, and interleukin 18 (IL-18).

## Materials and methods

### Animals

Quarter samples of the dairy cattle mammary gland parenchyma with predominance of the secretory tissue (MGST) were collected. The samples were obtained from 40 Polish Holstein–Friesian black-and-white cows differing in parity (1st to 4th lactation). Detailed information on the maintenance and feeding of the animals is described in Kościuczuk et al. [7]. All cows were culled in the last stage of lactation (approx. 280 days, SD = 25). Cows with mastitis were unsuccessfully treated with antibiotics several times, thus, they were slaughtered, but, at least one month after the last treatment. The samples assigned for the control group were taken from cows demonstrating problems with reproduction; i.e. all four quarters of the udder were free of bacteria. Samples taken from quarters exhibiting clinical symptoms of mastitis were not included in the analysis.

### Sampling and milk microbiological analysis

Samples of the mammary gland, approximately 1 × 1 × 5 cm in size, were taken from deep layers of parenchyma from each udder quarter immediately after slaughter. The samples were washed in ice-cold phosphate buffered saline (PBS, pH 7; Sigma Aldrich, Missouri, USA) to remove milk and remaining blood, and were then immediately flash-frozen in liquid nitrogen. Tissue samples were then stored at −80 °C for further analysis. The microbiological status of the cows’ udder samples was determined based on the detected microorganisms in milk. Approximately 20 mL of the foremilk samples were taken manually and aseptically two days before the slaughter from each udder quarter just before mechanical evening milking. Next, 100 µL samples of milk were streaked on a Columbia Agar supplemented with 5% sheep blood and Mannitol Salt Agar (bioMérieux, Craponne, France). The samples were incubated at 37 °C for 24–48 h. In addition to phenotypical and morphological assessment of bacterial colonies, identification was performed using catalase and coagulase production tests (tube test with rabbit plasma, Biomed, Warsaw, Poland). The biochemical properties of the bacteria strains were analyzed using the API test. Coagulase-positive bacterial isolates were additionally subjected to the SlidexStaph-Kit tests (bioMérieux, Craponne, France) to confirm *Staphylococcus aureus* identification.

Finally, 54 of the 160 MGST samples were selected for further analysis. The H group consisted of samples collected from cows without any pathogenic bacteria in their milk (*N* = 13 samples). The CoPS group consisted of samples collected from quarters infected with coagulase-positive staphylococci (*N* = 27 samples) (*S. aureus* only). The CoNS group included samples containing coagulase-negative staphylococci (*N* = 14 samples) with *Staphylococcus epidermidis* predominating. More information on the microbiological analysis is given by Bagnicka et al. [8]. The median somatic cell count (SCC) values were 1.3 × 10^6^ for the cows in the CoPS group and 1.2 × 10^6^ in the CoNS group at the last lactation. The cows with the whole healthy udder had a median SCC of 0.1 × 10^6^ during the whole of their last lactation. The health status history of the animals and treatment record was known from the herd management system.

### RNA isolation and its quantitative and qualitative assessment

RNA was extracted from 25 to 30 mg of frozen tissue samples using commercially-available RNeasy Mini kits (Qiagen, Hilden, Germany) with the addition of β-mercaptoethanol to lysis buffer (Merck, Darmstadt, Germany) and ssDNA/RNA Clean & Concentrator (Zymo Research, Irvine, USA) for RNA purification and concentration. Both procedures were performed according to the manufacturer’s protocols. The samples were analyzed for RNA integrity number (RIN) using the Bioanalyser 2100 (Agilent, Santa Clara, USA) with the RNA 6000 Nano LabChipKit (Agilent, Santa Clara, USA) and a NanoDrop 2000 spectrophotometer (Thermo Fisher Scientific, Waltham, Massachusetts, USA) according to the attached protocols. Only RNA samples with ratio A_260nm/280 nm_ between 1.9 and 2.2 and RIN > 7.0 were selected for further analysis.

### Reverse transcription and gene expression analysis

Reverse transcription was performed according to the Transcriptor First Strand cDNA Synthesis Kit (Roche, Basel, Switzerland) protocol. All samples obtained post-reaction were diluted to 50 ng/µL. The quantitative analysis of the relative number of transcripts of selected genes was performed by quantitative reverse transcription PCR (RT-qPCR) with a LightCycler480 (Roche). The name of the genes, the primer sequences, annealing temperature, the length of the amplicons, and GenBank accession numbers are listed in Table 1. Amplifications, according to the protocol, were carried out in three repeats with SYBR Green I (Roche) technology. RT-qPCR and the thermal profile were also set according to the manufacturer’s protocol: “LightCycler®480 SYBR Green I Master” (Roche). All RT-qPCR products were checked by gel electrophoresis in 2% agarose gel to confirm the presence of the gene of interest. Glyceraldehyde-3-phosphate dehydrogenase (*GAPDH*) and hypoxanthine–guanine phosphoribosyltransferase (*HPRT*) were used as reference genes (Table 2): these demonstrate stable expression, with an M-value in the MGST ranging between 0.6 and 0.3 [7].

**Table 1:** **Full names and abbreviations of gene names, primer sequences, length of amplicons, annealing temperature and access to GenBank, and/or source of chosen primers from the literature of the tested genes.**

Gene	Forward primer (F) Reverse primer (R)	Amplicon (bp)	Annealing temperature [°C]	GenBank Accession Number	References
*CXCL5*	(F) TGAGACTGCTATCCAGCCG	193	61	AF149249	[34]
	(R) AGATCACTGACCGTTTTGGG				
*TNFα*	(F) CGGTGGTGGGACTCGTATG	352	60	NM_173966.3	[35]
	(R) CTGGTTGTCTTCCAGCTTCACA				
*IL-18*	(F) GAAAATGATGAAGACCTGGAATCA	84	60	NM_174091.2	[35]
	(R) AACTTGGTCATTCAATTTCGTATGA				
IL-1β*	(F) AGAAAAGCCCGTCTTCCTGG	87	60	X12498.1	–
	(R) GGCTTTCTTTAGGGAGAGAGGG				
IL-6*	(F) TGCAGTCTTCAAACGAGTGG	156	60	NM_173923.2	–
	(R) TCTGACCAGAGGAGGGAATG				
*CCR1*	(F) CTGCTGGTGATGATTGTCTG	191	61°	NM_001077839	[36]
	(R) TGCTCTGCTCACACTTACGG				
*CCL2*	(F) CCCTCCTGTGCCTGCTACT	284	61°	NM_174006	[37]
	(R) TGCTCTGCTCACACTTACGG				

*Primers designed using Primer 3 0.4.0.

**Table 2:** **Full names and abbreviations of gene names, primer sequences, length of amplicons, annealing temperature and access to GenBank for selected reference genes**

Reference genes					
Gene	Forward primer	Reverse primer	Amplicon (bp)	Annealing temperature in real-time PCR [°C]	GenBank Accession Number
*GAPDH *(glyceraldehyde-3-phosphate dehydrogenase)	ACCACTTTGGCATCGTGGAG	GGGCCATCCACAGTCTTCTG	75	60°	U85042
*HPRT *(hypoxanthine–guanine phosphoribosyltransferase)	TGCTGAGGATTTGGAGAAGG	CAACAGGTCGGCAAAGAACT	154	60°	NW_001501830

*Primers designed using Primer 3 0.4.0.

### ELISA test

The concentrations of the eight analyzed cytokines were determined by ELISA (Enzyme-Linked Immunosorbent Assay). The materials used in the tests derived from the same tissue as for mRNA isolation: i.e. samples homogenized in PBS (pH 7; Sigma Aldrich, Missouri, USA) using tubes filled with silica beads (A&A Biotechnology, Gdynia, Poland). Commercially-available ELISA tests for CCR1, CCL2, CXCL5, TNFα, and IL-8 were purchased from the SunRed Bio company (Shanghai, China), while IL-1β, IL-6, and IL-18 were obtained from the Fine Biotech (Zhuan, China). All tests were conducted according to the manufacturers’ recommendations.

### Normalization of results, relative estimation of gene expression and statistical analysis

The results were normalized and relative gene expression calculated using the mathematical model described by Pfaffl [9], adapted by Kościuczuk et al. [7]. Since the effect of the lactation number was not statistically confirmed in the prior analysis and all animals were culled at the end of lactation, gene expression at the mRNA and protein levels was analyzed using one-way variance analysis (the fixed effect of the type of infection; i = 1, 2, 3); for this purpose, MIXED procedure was used with Tukey multiple range test with SAS (SAS/STAT, 2002–2012, v. 9.14). The normality of the distribution of transcript and protein concentrations was checked using the UNIVARIATE procedure (SAS/STAT, 2002–2012, v. 9.14). The mRNA levels were transformed into a natural logarithmic scale to normalize the distribution. The following cut-off points for significance were chosen: the values differ significantly at *p* ≤ 0.01 (A, B); the values differ significantly at *p* ≤ 0.05 (a, b); the values differ at the trend level at 0.05 < *p* < 0.1 (1, 2); and the values do not differ significantly at *p* ≥ 0.1.

## Results

**Figure 1:**
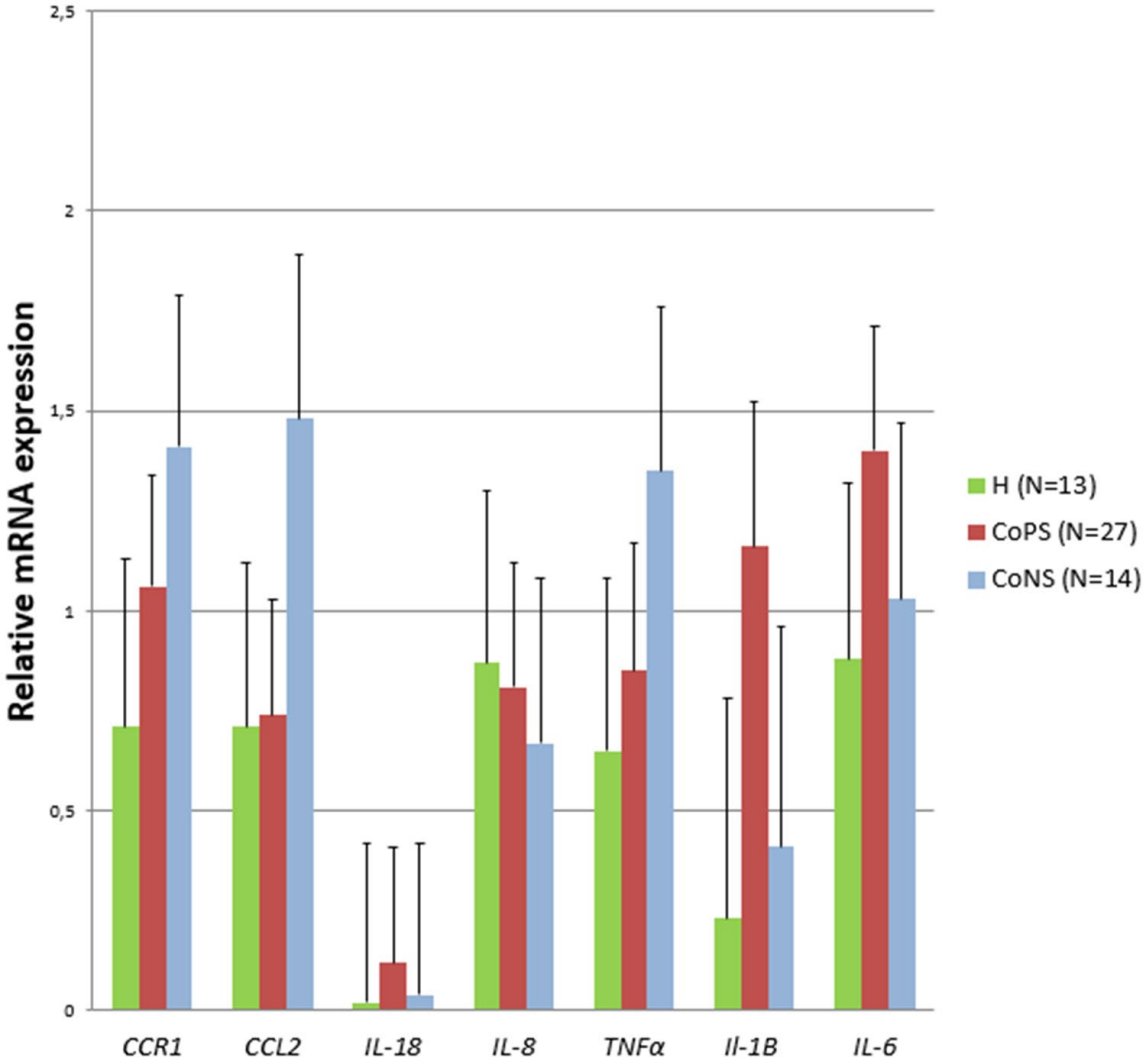
**The expression of the *CCR1, CCL2, IL-18, IL-8, TNFα, IL-1β, IL-6* genes in the udder parenchyma.** CoPS, coagulase-positive staphylococci; CoNS, coagulase-negative staphylococci; H, free from bacteria; *CCR1*, chemokine C–C motif receptor 1; *CCL2*, C–C motif chemokine 2; *IL-18*, interleukin-18; *IL-8*, interleukin-8; *TNFα*, tumor necrosis factor α; *IL-1β*, interleukin-1 β; *IL-6*, interleukin-6*.* The values within the same gene did not differ significantly (*p* ≥ 0.05).

**Figure 2:**
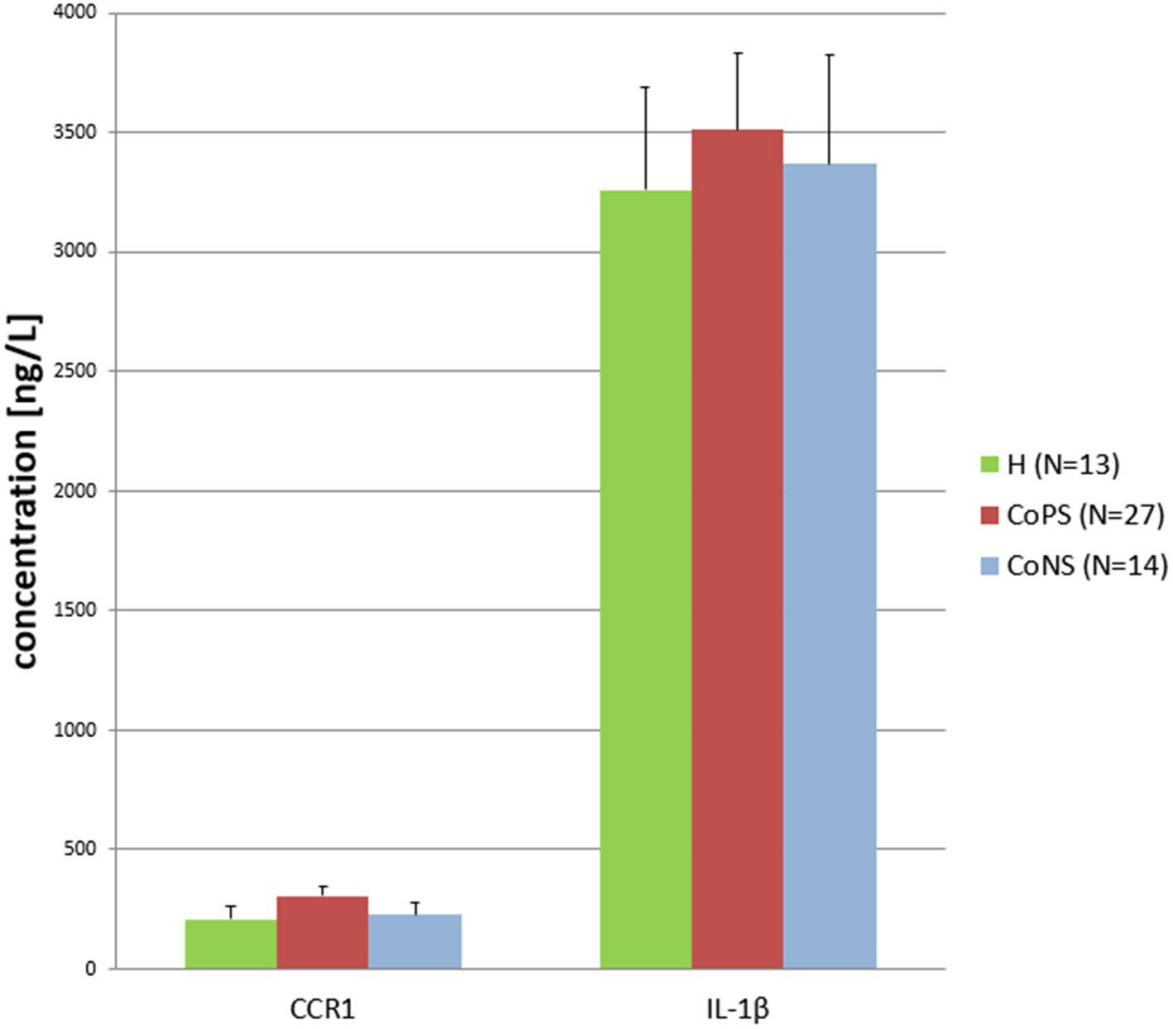
**The concentrations of CCR and IL-1β proteins in the udder parenchyma.** CoPS, coagulase-positive staphylococci; CoNS, coagulase-negative staphylococci; H, free from bacteria; CCR1, chemokine C–C motif receptor 1; IL-1β, interleukin-1Β. The values for the same protein did not differ significantly (*p* ≥ 0.05).

**Figure 3:**
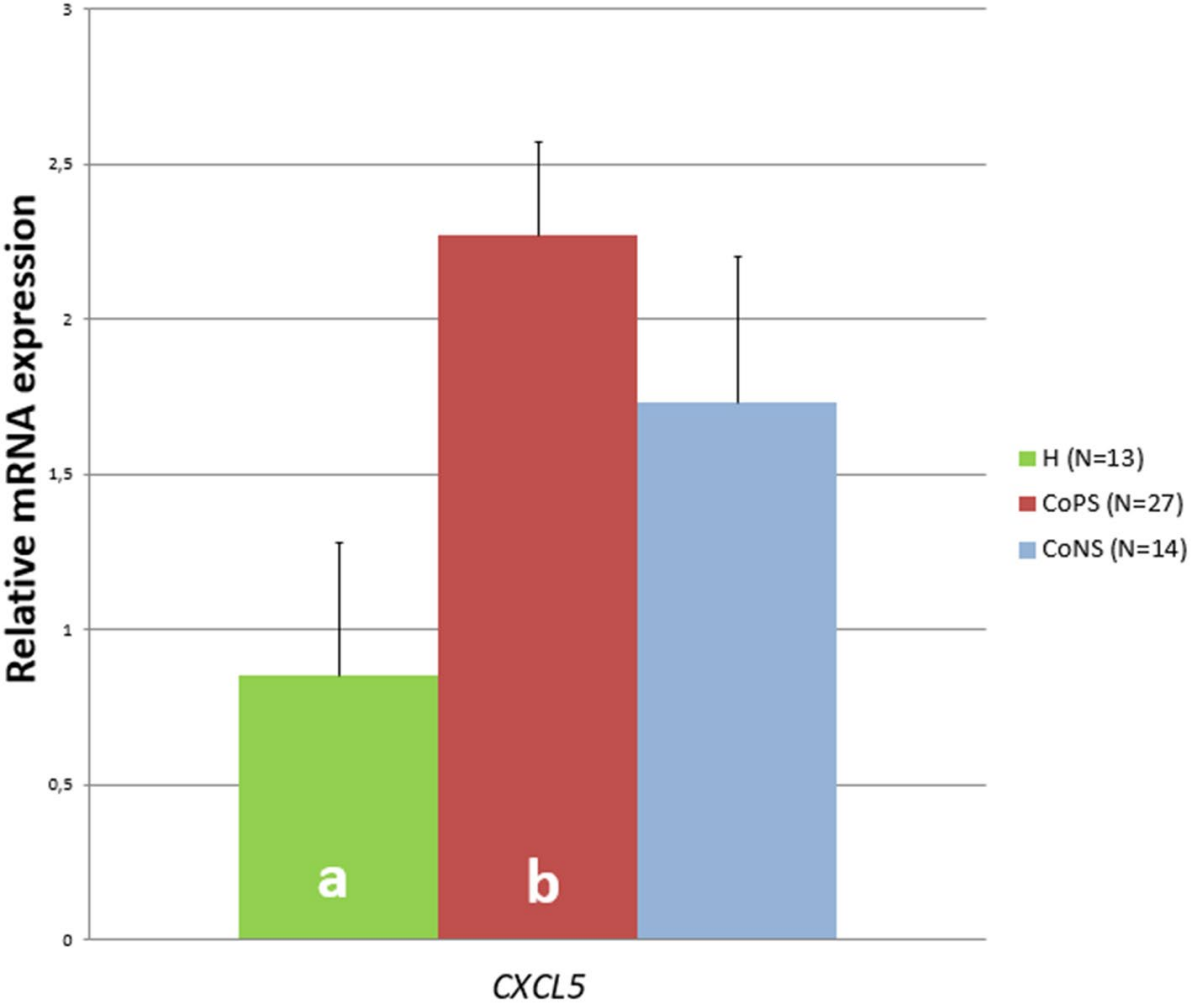
**The expression of the *CXCL5* genes in the udder parenchyma.** CoPS, coagulase-positive staphylococci; CoNS, coagulase-negative staphylococci*;* H, free from bacteria; *CXCL5*, C–C motif chemokine ligand 5; a, b—the values for the same gene with different letters differ at *p* ≤ 0.05.

**Figure 4:**
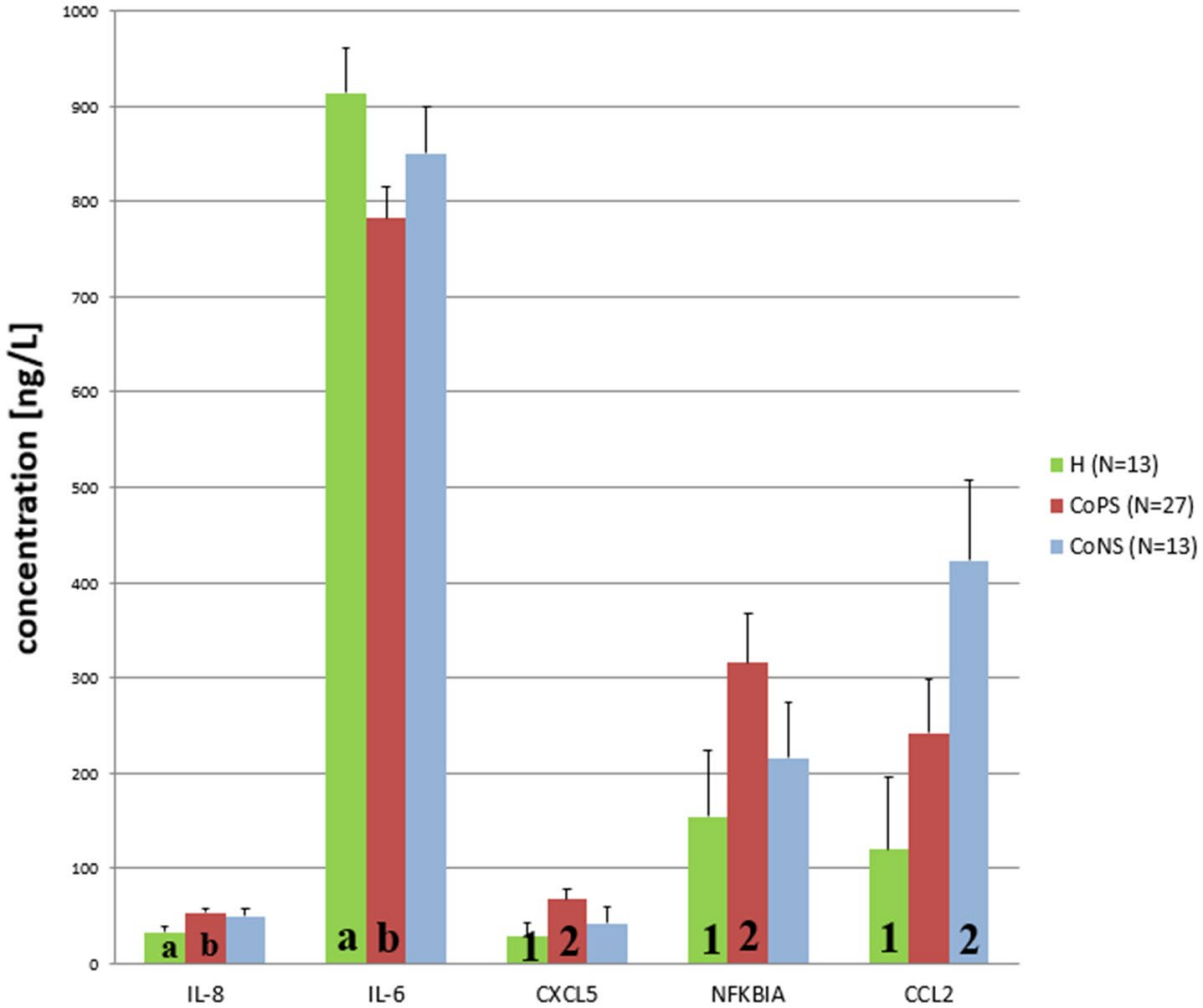
**The concentrations of IL-8, IL-6, CXCL5 and CCL2 proteins in the udder parenchyma.** CoPS, coagulase-positive staphylococci; CoNS, coagulase-negative staphylococci; H—free from bacteria; IL-8, interleukin-8; IL-6, interleukin-6; CXCL5, C–C motif chemokine ligand 5; CCL2, C–C motif chemokine 2; a, b—the values within the same protein with different letters differ at *p* ≤ 0.05; 1, 2—the values for the same protein with different numbers differ at 0.05 < *p* < 0.10.

No differences in the transcript levels of *CCR1*, *CCL2*, *TNFα*, *IL-1β*, *IL-6*, *IL-8*, or *IL-18* were found between groups. However, high variability was observed inside the groups, as indicated by the standard errors, suggesting that other unidentified features may have influenced the results (Figure 1). In addition, no differences were found regarding the concentrations of CCR1 and IL-1β protein (Figure 2). Moreover, the TNFα and IL-18 protein levels were below the detection level of the applied tests. Compared to the H tissue samples, the CoPS samples demonstrated higher *CXCL5* transcript levels (*p* ≤ 0.05), and higher protein concentrations of IL-8 and CXCL5 (*p* ≤ 0.05). In addition, compared to the H samples, the CoPS samples also demonstrated lower IL-6 levels (*p* ≤ 0.05) while the CoNS samples higher concentrations of CCL2 protein (0.05 < *p* < 0.1) (Figures 3 and 4).

## Discussion

CCR1, CCL2, TNFα, Il-1β, IL-6, IL8, and IL-18 are known to play key roles in acute mastitis, by activating lymphocyte, monocyte and neutrophil migration from the bloodstream to the site of inflammation [10]; however, their exact role during chronic inflammation remains unexplained. CCR1 is known to act as a receptor for the beta chemokines, such as CCL3 (Chemokine C–C motif ligand 3) and CCL7 (C–C motif ligand 7). Chemokines and their receptors play a crucial role in the signal transduction process used for recruiting immune system cells [11].

IL-1β plays a crucial role in the immune response during both acute and chronic inflammation; however, while IL-1β is secreted by various types of cells, such as monocytes and macrophages during the acute response, and is needed to activate the immune reaction during pathogen invasion, it also inhibits tissue damage during chronic inflammation. The exact mechanism of IL-1β secretion remains unclear. Until recently, it was thought that the protein was not produced in bovine mammary epithelial cells (MEC), despite the presence of its mRNA; this was attributed to the absence of two elements essential for the maturation and secretion of IL-1β by MEC: the inflammasome (the cytosolic protein complex regulating the activation of caspase-1) and caspase-1 (cleaving the pro-inflammatory cytokines IL-1β and IL-18 into their active form) [12]. Our present in vivo findings indicate that two of the eight studied genes, *CCR1* and *IL-1β*, demonstrate similar expression in healthy and infected tissues at both the mRNA and protein levels, suggesting that they did not participate in the udder defense against staphylococci during chronic mastitis. Although it does appear that IL-1β is produced in dairy cattle udder parenchyma, particularly in MEC, it is not possible to draw any firm conclusion on the in vivo expression of these genes at the mRNA and protein levels. Most studies of this topic have been conducted in vitro and usually only at the mRNA level; for example, Griesbeck-Zilch et al. [13] report elevated levels of *IL-1β*, *TNFα*, *IL-6* and *CCL5* transcripts 24 h after *S. aureus* challenge, but not *IL-8*; however, in contrast to our experiment, the majority of the presented results were obtained shortly after bacterial intrusion.

However, IL-18 has been observed on the surface of blood monocytes in healthy individuals, indicating that it is constitutively expressed in all cells in healthy humans and animals; the authors also propose that IL-18 appears to play a vital role in the synthesis of interferon-γ (IFN-γ) in T lymphocytes and NK cells, and classify it as an IL-1 cytokine. They also report that it demonstrates similar functions and activity to IL-1β [14]. In contrast to our results, Günther et al. [15] noted elevated *IL-18* expression at the mRNA level in cow mammary gland tissues during the acute phase of inflammation, i.e. three hours after artificial infection with *S. aureus*, compared to controls. In our present study, the presence of similar levels of *IL-18* mRNA and undetectable protein level (below ELISA test detection level) in samples from all groups, including controls, indicate that this cytokine is not involved in MGST defense. However, it is difficult to explain the lack of IL-18 protein products in MGST, especially in the light of previous observations that over 80% of the IL-18 precursor appears to remain unprocessed and is not secreted from the cell [14]. Therefore, more comprehensive studies at the mRNA and protein levels, or those examining non-coding RNA (ncRNA) expression, are needed to provide a clearer view of the function of IL-18 during acute and chronic inflammation.

Two other extensively-studied pro-inflammatory cytokines are IL-8, also known as chemokine CXCL8, and TNFα, whose main functions are recruiting neutrophils to the site of inflammation and maintaining the inflammatory response [16]. Although no significant differences were found between the analyzed groups for either *IL-8* or *TNFα* mRNA levels, higher IL-8 protein content was found in CoPS than H, and TNF*α* levels were below the detection limit in all samples. In our present study, all IL-8 protein levels were relatively low, even in the CoPS group. In contrast, Lee et al. [17] report that *IL-8* mRNA levels were elevated in milk somatic cells (MSC) after artificial intramammary infection with *E. coli* and *S. aureus*, with a faster and stronger inflammatory response to *E. coli* infection observed (75-fold increase after 16 h) compared to *S. aureus* (29-fold increase after 24 h). They stress that the elevated mRNA level returned very quickly (56 h for *E. coli* and 48 h for *S. aureus*) to baseline after the acute reaction i.e. 72 h after challenge. An even quicker reaction was observed by Strandberg et al. [18] in vitro: they note a rapid increase in the expression of *TNFα* and *IL-8*, as well as *IL-1β* and *CXCL6*, after two to four hours of stimulation with *S. aureus*, with these levels quickly decreasing to baseline after eight to 16 h. They postulate that this temporary/short-term growth in cytokine expression may explain why staphylococcal infection tends to take more of a chronic than an acute course. A combined in vitro and microarray study by Xiu et al. [19] confirmed that levels of *IL-8* mRNA, but not *TNFα* mRNA, were elevated in MEC after artificial challenge by *S. aureus.* However, microarray analysis of MGST identified elevated *IL-8* and *TNFα* transcript levels following staphylococcus infection, with the results for *Il-8* being confirmed by RT-qPCR analysis [7]. In contrast to those results, but in line with our present findings, artificial infection with *S. aureus* did not appear to affect the mRNA levels of *TNFα* and *IL-8* in cow MSC, and the IL-8 and TNFα levels were low or undetectable in milk [20]. Similar results regarding *TNFα* transcript level have been presented by Günther et al. [15] and Griesbeck-Zilch et al. [13]; however, they did not show any changes in *IL-8* expression.

Similarly to the present study, Bannerman et al. [20] were not able to detect IL-8 and TNFα concentration in MSC of cows infected with *S. aureus*; they propose using an ELISA test with higher sensitivity in further studies. However, it is also possible that *S. aureus* can disrupt the translation of these two key cytokines using its own defense mechanisms, perhaps via regulation of ncRNA expression: *S. aureus* has been proposed to influence the activity of miR-99b, an ncRNA that targets the *TNFα* gene [21], as is *Mycobacterium tuberculosis* [8]. Further studies indicate that increased levels of *IL-8* and *TNFα* transcripts do not necessarily imply an increased concentration of their proteins and vice versa [17, 22]. An in vitro study by Lahouassa et al. [10] found elevated mRNA and protein expression of TNFα and IL8 in bovine MEC following *S. aureus* stimulation, with the elevated protein levels only being observed in vitro, and not in mastitic milk. However, further research is needed in this area, as IL-8 concentration has been found to be elevated in this current study using chronic mastitic model in contrary to Lahouassa et al. [10]. Even so, the combination of stable *TNFα* mRNA expression with low TNFα protein levels suggests that TNFα does not play an important role in chronic mastitis, and that this may be attributed to the influence of epigenetic regulation.

On the contrary, several miRNA are known to target the *TNF* gene but only one influences *IL-8* [23]. A number of studies have found that an elevated level of *TNFα* transcripts does not coincide with high protein concentration in MEC [12]. Previous in silico analysis of cow mammary tissue based on an interaction network comprising common target genes shared between CoPS and H groups found *TNFα* gene expression may be regulated by four miRNA during CoPS infection, namely bta-miR-21-5p, bta-miR-146b, bta-miR-155 and bta-miR-223 [8]. A study of bovine MEC identified the presence of less than 50 pg/mL of TNFα protein; it is probably essential for mammary gland cells to block high levels of TNFα production since TNFα inhibits casein synthesis [12].

One of the genes that demonstrates higher transcript and protein expression in the CoPS than the H group was *CXCL5* (0.05 < *P* < 0.10). During udder inflammation, CXCL5 stimulates neutrophil-directed chemotaxis and influences the recruitment of lymphocytes, mast cells, granulocytes, and monocytes to the site of infection [24]. In humans, this protein is expressed concomitantly with IL-8 as a response to Il-1β or TNFα stimulation [25]. Xiu et al. [19] report an upregulated level of *CXCL5* transcripts in bovine epithelial cells after *S. aureus* infection. Similarly, elevated *CXCL5* gene expression was found in bovine MGST in vivo using a chronic mastitis model by Kościuczuk et al. [26] and in a microarray-based in vitro study by Gilbert et al. [12]. The increased expression of *CXCL5* found in our study, and in previous studies, may suggest that CXCL5 participates in the immune response regardless of the course of inflammation, i.e. acute or chronic, by recruiting different types of immune cells to the site of inflammation. It may be responsible for the elevated SCC observed in the milk from udders infected with staphylococci despite the lack of any clinical signs of mastitis. Although the production of CXCL5 appears to be stimulated by IL-1β or TNFα in humans [25], our present findings do not confirm this in bovines: no significant differences in IL-β concentration were found between groups, while TNFα protein appeared to be absent from MGST, regardless of the state of udder health. Even so, our present findings indicate the presence of elevated IL-8 concentrations in the CoPS group, together with elevated CXCL5 levels. Therefore, it appears that in bovine mastitis, both cytokines are regulated in a different manner than in human cells.

Il-6 is a pro-inflammatory cytokine that is responsible mainly for the acute phase reaction at the very beginning of inflammation (e.g., fever) caused by both coliforms and staphylococci. It is involved in antigen recognition by T lymphocytes and acts as an activator of B lymphocyte differentiation [27]. IL-6 stimulates the production of acute phase proteins, mainly by hepatocytes [20], as well as hematopoiesis. It plays a key role in bovine mastitis, with its concentration in bovine MEC being elevated six hours and 24 h after challenge with *S. aureus* compared to one hour after challenge [13]. Günther et al. [15] found IL-6 to be the only cytokine to demonstrate increased mRNA expression in MEC after *S. aureus* challenge, as indicated by microarray analysis, while RT-qPCR analysis found it to increase only three-fold compared to controls. In addition, Ingenuity Pathway Analysis based on data from a series of studies [28] found IL-6 to dominate the *S. aureus* functional network. In addition, IL-6 concentrations in cow milk were elevated by subclinical inflammation caused by four species of staphylococci compared to healthy controls [27]. However, it was noted that the protein concentration fell as the disease progressed. In contrast, our present findings indicate no differences in transcript level between groups. In addition, IL-6 concentration was reduced in the CoPS group compared to the H controls; this was probably associated with the fact that our present study examined chronic mastitis while previous studies only concerned acute inflammation, and that most of them were conducted in vitro on cultured MEC. However, *Il-6* is known to be targeted by miR-125-5p, miR-155-5p and miR-939-5p, as indicated by DIANA tools TarBase v.8 analysis [29]. Of these, miR-155 has been found to be elevated in CoPS tissue compared to healthy controls, and this could be the explanation for the decreased IL-6 production since miRNA mainly inhibited translation of its target gene [8].

Similar to the pro-inflammatory cytokines mentioned above, CCL2 (*alias* monocyte chemoattractant protein 1; MCP-1), recruits monocytes, memory T cells, and dendritic cells to the site of inflammation [30] as well as eosinophils, and basophils [15]. Although no differences in *CCL2* transcript levels were found between the groups analyzed in our research, the CCL2 concentration was higher in the CoNS group than the H group: in fact, it was the sole pro-inflammatory factor with an elevated concentration in the CoNS group. In other studies, CCL2 concentration was found to be elevated in bovine MEC following *S. aureus* stimulation, and the authors suggest that the initial inflammatory response demonstrated in mammary gland to *S. aureus* infection may be associated with LTA, this being the main component of the Gram-positive bacteria cell wall and one that can induce chemokine genes [30]; however, these findings appear true only for *S. aureus*, as LTA is found in the cell walls of all Gram-positive bacteria, i.e. those present in both the CoPS and CoNS samples, and no differences were observed between CoPS and H in the present study. The dissimilarity between findings described by Kiku et al. [30] and those of our present study may be accounted for essential differences in staphylococcal modes of infection: CoNS and CoPS use different approaches to survive and propagate inside host cells. While CoPS have a vast set of properties facilitating invasion and survival, and even allowing them to remain undetected inside cow udder cells, CoNS may be detected and removed more easily from the organism, thus yielding a different immune system reaction. Our results suggest that CCL2 may play an important role in the host response to CoNS infection, but not during chronic inflammation caused by CoPS.

In contrast to our results, Gilbert et al. [12] report elevated CCL2 mRNA levels in bovine MEC three hours and six hours after *S. aureus* challenge; however, the study was based on an acute response model. Günther et al. [15] also note a four-fold increase in *CCL2* transcripts in MEC 24 h after *S. aureus* challenge. In addition, an earlier microarray analysis [7] found *CCL2* gene expression to be upregulated in udder samples of chronic mastitis caused by CoPS; however, this was only true in samples derived from cows in their first or second lactation and not from older cows. In the CoNS group, upregulated expression was observed only in samples derived from cows in their third or fourth lactation. Despite this, any conclusions should be drawn tentatively, as the microarray analysis only addresses *CCL2* mRNA expression during chronic udder inflammation, not protein expression: it can only be supposed that chronic CoNS infections should not be regarded as less harmful than CoPS. CCL2 is a pro-inflammatory cytokine which recruits leukocytes to the site of inflammation; it also triggers another cytokine cascade, which in turn recruits immune cells similarly to CoPS.

In addition, no significant differences were found between groups regarding mRNA expression while a higher concentration of CCL2 protein was observed in the CoNS group. This may suggest that CoNS may enhance CCL2 translation and protein production by epigenomic changes. In addition, it is possible that during chronic mastitis, CoPS may stimulate the MGST immune response in different ways to CoNS since more proinflammatory factor genes were found to be upregulated in the CoPS group. DIANA tolls TarBase v.8 analysis suggests that CCL2 may be a target gene for only hsa-miR-128-3p, which was not found to be differentially expressed in either CoPS or CoNS compared to H in our previous study [8].

In the present study, some genes and/or translation processes were not expressed during chronic mastitis, despite the presence of bacteria in MGST. This may be due to the host attempting to avoid self-destruction, despite the presence of acute inflammation. Many immune system mechanisms, such as the release of reactive oxygen species from lymphocytes, macrophages and neutrophils, also have cytotoxic effects on host cells in the first hours after bacterial invasion [31], and the host reaction is often so intense during acute phase response that the tissues are irreversibly damaged, thus allowing further bacterial infection [32]. During the acute phase reaction, i.e. the first 48 h after infection, the concentration of acute phase proteins, these being the first line of pro-inflammatory mediators activated after pathogen intrusion, rises to 100-fold higher than in healthy individuals; later, when the acute phase moves to a chronic one, this value falls to approximately a few tenfold higher [33].

The differences observed between mRNA and protein levels of some studied cytokines (e.g., *CCL2*, *IL-8*, *IL-6*) may be caused by epigenetic mechanisms, such as short non-coding RNA or DNA methylation; however, many miRNA and their target genes remain unknown, and it is impossible to explain the mechanisms in detail. In addition, a range of regulatory processes work together to maintain the cell or organism in continually changing conditions, and the control of gene expression is subject to both the external and internal environment of the organism [29]. Although several studies have examined the inflammatory response of MGST to bacterial infections, most were carried out on animals infected experimentally or used in vitro procedures examining short periods of acute inflammation. Many immune response mechanisms remain unclear. Our present study on naturally-infected cows shows that CCL2, CXCL5, IL-8, IL-6 were also produced in MGST during chronic mastitis, and that the transcript levels of *CCR1*, *CCL2*, *TNFα*, *Il-1β*, *IL-6*, *IL-8* and *IL-18*, and the protein concentrations of CCR1, TNFα, Il-1β and IL-18 remained stable and did not differ between infected and non-infected tissues.

The proteins crucial for acute mastitis (IL-18, Il-1β, TNFα, CCL2, CCR1) demonstrate low expression. This may suggest that during the chronic stage of the disease, the organism stops producing pro-inflammatory cytokines, probably to protect the host tissues against their damage during prolonged infection. This may mean that distinct mechanisms, not fully known yet, are triggered during chronic mastitis and not during acute infection. Differences observed in mRNA and protein expression are likely due to the post-transcriptional and/or post-translational regulations. Therefore, it is crucial to study gene expression at both the mRNA and protein levels.

## Abbreviations

CCL2: chemokine C–C motif ligand 2
CCL3: chemokine C–C motif ligand 3
CCL7: chemokine C–C motif ligand 7
CCR1: chemokine C–C motif receptor 1
CoNS: coagulase-negative staphylococci
CoPS: coagulase-positive staphylococci
CXCL5: chemokine C–C motif ligand 5
ELISA: Enzyme-Linked Immunosorbent Assay
GAPDH: glyceraldehyde-3-phosphate dehydrogenase
HPRT: hypoxanthine–guanine phosphoribosyl transferase
IL-18: interleukin 18
IL-1β: interleukin 1β
IL-6: interleukin 6
IL-8: interleukin 8
KEGG: Kyoto Encyclopedia of Genes and Genomes
LPS: lipopolysaccharide
LTA: teichoic acid
MCP-1: monocyte chemoattractant protein 1
MEC: mammary epithelial cell
MGST: mammary gland secretory tissue
NK: natural killer cells
PAMP: pathogen-associated molecular patterns
PBS: phosphate buffered saline
PRR: pathogen recognition receptors
RIN: RNA integrity number
SCC: somatic cell count
TLR: toll-like receptor
TNFα: tumor necrosis factor α
